# Complementary monoclonal antibody-based dot ELISA for universal detection of H5 avian influenza virus

**DOI:** 10.1186/1471-2180-10-330

**Published:** 2010-12-30

**Authors:** Fang He, Retno D Soejoedono, Sri Murtini, Michael Goutama, Jimmy Kwang

**Affiliations:** 1 Animal Health Biotechnology, Temasek Life Sciences Laboratory, National University of Singapore, Singapore, 117604; 2 Faculty of Veterinary Medicine, Depart of Infectious diseases & Vet. Public Health, Division of Microbiology, Institute Pertanian, Bogor, Indonesia; 3 Tridel Biosciences International Pte Ltd, Singapore; 4 Department of Microbiology, Faculty of Medicine, National University of Singapore, Singapore

## Abstract

**Background:**

Rapid diagnosis and surveillance for H5 subtype viruses are critical for the control of H5N1 infection.

**Results:**

In this study, H5 Dot ELISA, a rapid test for the detection of avian H5N1 influenza virus, was developed with two complementary H5 monoclonal antibodies. HA sequencing of escape mutants followed by epitope mapping revealed that the two Mabs target the epitope component (189^th ^amino acid) on the HA protein but are specific for different amino acids (189Lys or 189Arg). Gene alignment indicated that these two amino acids are the most frequent types on this position among all of the H5 AIV reported in GeneBank. These two H5 Mabs were used together in a dot ELISA to detect H5 viral antigen. The detection limit of the developed test for multiple clades of H5N1 viruses, including clades 0, 1, 2.1, 2.2, 2.3, 4, 7, and 8, was less than 0.5 hemagglutinin units. The specificity of the optimized dot ELISA was examined by using 100 H5 strains, including H5N1 HPAI strains from multiple clades, 36 non-H5N1 viruses, and 4 influenza B viruses. No cross-reactivity was observed for any of the non-H5N1 viruses tested. Among 200 random poultry samples, the test gave 100% positive results for all of the twelve RT-PCR-positive samples.

**Conclusions:**

Considering that the test is convenient for field use, this H5 Dot ELISA can be used for on-site detection of H5N1 infection in clinical or environmental specimens and facilitate the investigation of H5N1 influenza outbreaks and surveillance in poultry.

## Background

Influenza A virus is classified into subtypes H1 to H16 and N1 to N9 based on the antigenic specificity of hemagglutinin (HA) and neuraminidase (NA). The 16 HA subtypes of the influenza viruses found in aquatic birds act as the carrier (reservoir) of all avian influenza virus A [1]. Only two influenza A subtypes (H1N1 and H3N2) are currently circulating in the human population, while H5 and H7 are the most malignant, causing death in avian species [2]. The emergence of the H5N1 highly pathogenic avian influenza (HPAI) virus caused highly contagious and deadly disease outbreaks in poultry in several Asian countries, including China, Indonesia, Cambodia, Japan, Korea, Laos, Thailand, and Vietnam [3-5]. Recently, the H5N1 virus has been shown to spread incessantly to many regions all over the world [6]. Most of these outbreaks were confined to poultry, but the virus was reported to be transmitted to humans in a few countries and most of these cases lead to death in infected human. Despite the comparatively small number of human cases, this situation warrants careful monitoring. Of foremost concern is the risk that conditions in parts of Asia could give rise to an influenza pandemic [7]. As of August 2010, there have been totally 505 cases of confirmed H5N1 infection in humans, resulting in 300 fatalities [8].

Rapid and sensitive laboratory and field tests for the diagnosis of H5N1 HPAI infection are essential for disease control [9]. Conventional laboratory methods for H5N1 virus detection include virus isolation in embryonated eggs or Madin-Darby canine kidney (MDCK) cells, followed by subsequent HA and NA subtype identification using serological methods [10,11]. Molecular detection methods such as reverse transcriptase PCR (RT-PCR) have been widely applied for the laboratory diagnosis of influenza infections and HA subtype identification [12,13]. However, these methods are technically demanding and time consuming, or requiring high level biosafety facility. Therefore, antigen detection based on serologic methods has repeatedly shown its value to diagnose various infectious diseases.

The development of a panel of broad spectrum H5-specific monoclonal antibodies used in rapid antigen tests allows to differentiate H5 subtype from other HA types in the field. Detection of H5 antigen provides strong evidence of H5 avian influenza virus infection [14]. Monoclonal antibody (Mab) based diagnostic antigen detection tests for H5 AIV have been reported. Monoclonal antibodies are a homogenous population of antibodies, derived from a single antibody-producing cell whereby all antibodies produced are identical and of the same specificity for a given epitope [15]. The specificity of these Mabs responses provides a basis for an effective diagnostic reagent [16]. However, as an RNA virus, AIV tends to change its antigenic structure during evolution. Antigenic drift leads to hemagglutinin variants within each HA subtype from different globe regions at different times. Certain Mabs that specifically target a given HA epitope of one type AIV, may not be able to recognize other AIV strains with a mutated antigenic epitope even if such a mutation is slight. Therefore, using one single Mab for H5 AIV antigen detection, in most cases, will not cover all the H5 subtype AIV circulating world around. Here we report the development of an antigen-capture dot ELISA based on a pair of Mabs targeting the same epitope on H5, however, by two different and dominant amino acids respectively, in an attempt to make a universal H5 AIV rapid detection test.

## Results

### Identification of monoclonal antibodies recognizing complementary epitopes on H5 hemagglutinin

A panel of Mabs against influenza hemagglutinin was screened for efficient detection of different strains of H5N1 viruses. Based on the results of the HI assay, Mabs 6B8 and 4C2 were chosen for further studies due to their high HI activity (Table 1) against a wide range of rescued reassortant viruses from different clades. Both Mabs were found to be of the IgM isotype. After the virus neutralizing activity has been confirmed (data not shown), the amino acids involved in forming the epitopes of the Mabs were analyzed using escape mutant analysis. All HA amino acid numbering in this work uses H5 numbering excluding the signal peptide. Upon sequencing escape mutants from 3 different parental strains (Table 2), a few mutant clones of Mab 6B8 showed mutations at either Lys189 or Asn155, while clones of Mab 4C2 presented mutations at Arg189, Ser155 or Asn155. The results indicated that the 189^th ^and 155^th ^amino acids were involved in the epitopes of both Mabs, but in different forms. Mab 6B8 is able to bind to H5 with either Lys189 or Asn155 independently. Mab 4C2 binds to Arginine on 189^th ^amino acid of H5, and it recognizes both Serine and Asparagine at position 155.

**Table 1:** Hemagglutination Inhibition (HI) titers of the Mab 6B8 and 4C2 (1 mg/ml) against H5N1 influenza viruses.

Virus	Clade	6B8	4C2
A/Indonesia/CDC669/06	2.1	<8	512

A/Indonesia/CDC594/06	2.1	256	128

A/Vietnam/1203/04	1	512	<8

A/Hongkong/156/97	0	256	128

A/turkey/Turkey1/05	2.2	256	128

A/Anhui/1/05	2.3	256	<8

A/goose/Guiyang/337/06	4	128	128

A/chicken/Shanxi/2/06	7	256	256

A/chicken/Henan/12/04	8	256	128

**Table 2:** Amino acids on HA of H5N1 influenza viruses recognized by Mab 6B8 and 4C2, which identified in the comparison between parental virus and cloned escape mutants.

Virus	6B8	4C2
A/Indonesia/CDC669/06 (155Ser, 189Arg)	--	189Arg, 189Arg155Ser

A/Indonesia/CDC594/06 (155Asn, 189Arg)	155Asn	155Asn

A/Vietnam/1203/04 (155Ser, 189Lys)	189Lys	--

The number indicated the amino acid position in H5 HA. The amino acid type on the position was shown after the number. --: The Mab does not react with the parental virus.

In order to determine the significance of the two epitopes of 6B8 and 4C2, the protein polymorphism of H5 was studied, taking into account all H5 sequences in the NCBI database. As shown in the Table 3, among both avian and human H5N1 strains, Arg and Lys appear in more than 98.4% of H5N1 strains in the 189^th ^amino acid, while Asn and Ser are the most dominant amino acids in the position 155. This finding indicated that the Mab pair, which can cover the two epitopes, is able to recognize more than 98.4% of H5N1 strains in the database. Based on this, Mabs 6B8 and 4C2 were thought to have good potential for being used in combination to detect H5N1 infections.

**Table 3:** The protein polymorphism of H5 on 155th and 189th amino acid.

	Human H5N1		Avian H5N1	
155^th ^aa	Asn 34.4%	Ser 63.4%	Asn 50.6%	Ser 43.2%

189^th ^aa	Arg 64.3%	Lys 34.7%	Arg 43.3%	Lys 55.1%

To further ascertain this prediction, an antigen capture ELISA was performed, and the two Mabs were found to recognize complementary epitopes and react with all the H5N1 viruses from different clades available in our laboratory (Table 4). Hence, it was concluded that the Mab 6B8 and 4C2 complemented each other and were a good pair to use in rapid antigen detection of H5 influenza.

**Table 4:** Evaluation of the specificity and sensitivity of Mab 6B8 and 4C2 against H5 subtype influenza viruses.

Virus	Clade for H5N1 or Subtype	Absorbance reading in AC-ELISA (OD_490_)^a^	Detection Limit (HA unit) in dot ELISA	Detection Limit (HA unit) in dot ELISA (Rockeby)
A/Hong Kong/156/97	0	1.323	0.25	1

A/Hong Kong/213/03	1	0.965	0.5	1.5

A/Vietnam/1203/04	1	1.235	0.25	1

A/Indonesia/CDC7/06	2.1.1	1.149	0.25	1.5

A/Indonesia/CDC594/06	2.1.2	1.326	0.125	1

A/Indonesia/CDC370/06	2.1.3	0.963	0.5	1.5

A/Indonesia/CDC523/06	2.1.3	1.234	0.25	1.5

A/Indonesia/CDC326/06	2.1.3	1.062	0.25	1

A/Indonesia/CDC669/06	2.1.3	1.085	0.5	1.5

A/turkey/Turkey1/05	2.2	1.247	0.25	0.5

A/barheaded goose/Qinghai/12/05	2.2	1.096	0.25	1

A/Nigeria/6e/07	2.2	0.954	0.5	1.5

A/Anhui/1/05	2.3	0.853	0.5	1.5

A/chicken/Nongkhai/NIAH400802/07	2.3	1.047	0.5	1

A/Vietnam/HN31242/07	2.3	1.247	0.5	1.5

A/goose/Guiyang/337/06	4	1.193	0.25	0.5

A/chicken/Shanxi/2/06	7	1.085	0.5	1

A/chicken/Henan/12/04	8	0.975	0.5	1.5

A/Puerto Rico/8/34	H1N1	0.052	-	1

A/Singapore/TLL10/2009	H1N1	0.046	-	1

A/Singapore/TLL54/2009	H1N1	0.058	-	0.5

A/duck/Nanchang/4-184/2000	H2N9	0.056	-	1

A/chicken/Singapore/02	H3N2	0.061	-	1

A/Netherlands/219/03	H7N7	0.059	-	1.5

^a^Values represent the mean absorbance from triplicate wells.

### Combination of monoclonal antibodies in H5 dot ELISA

Different concentrations of Mabs were used before confirming the optimal concentration in a prototype rapid test. 1 ug each of 6B8 and 4C2 were immobilized on a nitrocellulose membrane and serve as capturing antibodies, The sample was lysed with 400 ul lysis buffer and loaded on the membrane with a filter device. The H5N1 virus captured by the immobilized H5-specific antibodies is detected using a mixture of Mab 6B8 and 4C2 labeled with horseradish peroxidase. The entire test procedure is completed within 30 minutes [14].

### Determination of analytical specificity and sensitivity

The specificity of the H5 dot ELISA was tested with a total of 100 HPAI H5 strains isolated from humans and avian species and 40 non-H5 subtype influenza virus strains from different regions and years, including 26 seasonal influenza virus strains (H1N1, H3N2, and B subtypes) and 2 pandemic influenza virus strains circulating in humans. Viruses of H5 or HA subtypes not available in our laboratory were rescued by reverse genetics with the six internal genes from A/Puerto Rico/8/34. The reactivity and specificity of the H5 dot-ELISA were examined with 200 ul of PBS containing the H5 strains adjusted to an HA titer of 8. Non-H5 viruses with HA titers of 16 were used in order to eliminate false-positive results. Virus strains listed in Table 5 and 6 were tested in the laboratory and the rest strains were studied at the sites of those virus donors. The dot ELISA rapid test with 4C2 and 6B8 can successfully detect all the 100 H5 virus strains from different clades, covering clades 1, 2.2, 2.3, 0, 7, 4, and 8, and representative H5 Indonesia isolates, which belong to clade 2.1. No cross-reactivity was observed for any of the non-H5 subtype viruses tested. Other avian viruses such as Newcastle Disease (ND), Infectious Bursal disease (IBD), were also tested to be negative with the H5 dot ELISA.

**Table 5:** List of H5N1 strains tested in the laboratory

Virus	Clade
A/Hong Kong/213/03	1

A/Vietnam/1203/04	1

A/muscovy duck/Vietnam/33/07	1

A/Indonesia/CDC1031/07	2.1

A/Indonesia/CDC7/06	2.1

A/Indonesia/CDC326/06	2.1

A/Indonesia/CDC329/06	2.1

A/Indonesia/CDC370/06	2.1

A/Indonesia/CDC390/06	2.1

A/Indonesia/CDC523/06	2.1

A/Indonesia/CDC594/06	2.1

A/Indonesia/CDC595/06	2.1

A/Indonesia/CDC597/06	2.1

A/Indonesia/CDC610/06	2.1

A/Indonesia/CDC623/06	2.1

A/Indonesia/CDC644/06	2.1

A/Indonesia/CDC669/06	2.1

A/Indonesia/TLL01/06	2.1

A/Indonesia/TLL02/06	2.1

A/Indonesia/TLL177/06	2.1

A/Indonesia/TLL298/06	2.1

A/Indonesia/TLL485/06	2.1

A/Indonesia/TLL530/06	2.1

A/Indonesia/TLL535/06	2.1

A/Indonesia/TLL540/06	2.1

A/Indonesia/TLL561/06	2.1

A/Indonesia/TLL565/06	2.1

A/Chicken/Indonesia/TLL101/06	2.1

A/Duck/Indonesia/TLL102/06	2.1

A/turkey/Turkey1/05	2.2

A/barheaded goose/Qinghai/12/05	2.2

A/Nigeria/6e/07	2.2

A/muscovy duck/Rostovon Don/51/07	2.2

A/chicken/Nongkhai/NIAH400802/07	2.3

A/Jiangsu/2/07	2.3

A/Anhui/1/05	2.3

A/Vietnam/HN31242/07	2.3

A/Vietnam/HN31242/07	2.3

A/Hong Kong/156/97	0

A/goose/Guiyang/337/06	4

A/chicken/Shanxi/2/06	7

A/chicken/Henan/12/04	8

**Table 6:** List of non-H5N1 tested in the laboratory

Virus	Subtype
A/Singapore/TLL10/2009	H1N1

A/Singapore/TLL54/2009	H1N1

A/Puerto Rico/8/34	H1N1

A/duck/Nanchang/4-184/2000	H2N9

A/Chicken/Singapore/Singapore/02	H3N2

A/Chicken/Singapore/Singapore/92	H4N1

A/Common Iora/Indonesia/F89/11/95	H5N2

A/chicken/Singapore/97	H5N3

A/shorebird/DE/12/04	H6N8

A/Chicken/Singapore/94	H7N1

A/Netherlands/219/03	H7N7

A/duck/Yangzhou/02/05	H8N4

A/Chicken/Singapore/Singapore/98	H9N2

A/Mandarin Duck/Singapore/Singapore/93	H10N5

A/pintail/Alberta/49/03	H12N5

A/gull/Maryland/704/1977	H13N6

A/herring gull/Delaware/712/1988	H16N3

The analytical sensitivity of the H5 Dot ELISA was determined against 18 strains of H5 virus belonging to the major genetic groups (clades or subclades) diluted serially. As shown in Table 4, the detection limit of the test varied from 0.5 to 0.125 HA units/200 ul of sample. The detection limit of the commercial kit for influenza A virus detection (Rockeby) was determined to be 200 ul of sample containing at least 1.5 HA titer of virus.

### Performance of H5 dot ELISA in the detection of variant H5N1 Indonesia strains in poultry samples relative to RT-PCR

The dot ELISA test was further evaluated with poultry samples. The swabs from birds infected with H5N1 virus can secrete virus of titer higher than l0^8 ^EID_50_/ml. Samples were serially diluted 10 times from 10^-1 ^to 10^-4 ^with PBS and tested by the dot ELISA kit to determine the detection limit for swabs. The sensitivity test indicated that the dot ELISA kit was able to detect the presence of virus at a concentration down to 10^5 ^EID_50_/ml in swabs, suggesting the test can be used for the detection of H5 infection in sick birds.

From 150 samples taken from clinically healthy birds, one sample was found to be positive with the test. The same sample was confirmed to be the only positive swab among the 150 samples in RT-PCR with H5 specific primers. 50 tracheal swabs obtained from sick birds were also tested with both dot ELISA and RT-PCR (Table 7). The results with the dot ELISA showed that nine samples were positive for H5 infection. The same result was observed from the verification with RT-PCR.

**Table 7:** Results of detection of H5 virus in random tracheal swabs using the dot ELISA kit and RT-PCR

Source of sample (area)	Source of animal	Clinically condition of animal	Number of samples	Result of test using		Sensitivity (%)
					
				Dot ELISA	RT-PCR primer H5	
Makasar	Native chicken	Healthy	50	1	1	100

Bogor	Layer chicken	Healthy	50	1	1	100

Bogor	Broiler chicken	Healthy	50	1	1	100

Bogor	Chicken and duck	Sick	50	9	9	100

As shown in Table 8, specificity test using various H5N1 viruses from several years and areas in Indonesia showed that the ELISA kit is 90% specific compared with RT-PCR using H5 primers, but 100% specific compared to HA2 primer. This indicates that the dot ELISA kit is able to detect H5N1 as long as the virus did not undergo a genetic mutation in their HA genes. Taken together, these findings indicate that the dot ELISA kit is suitable for specific early detection of H5 virus infection in avian species.

**Table 8:** Results of detection of H5 strains from poultry samples using the dot ELISA kit and RT-PCR

Source of isolates	Source of area	Time of first isolation	Dot ELISA	H5 primer pair one	H5 primer pair two	HA2 primer pair
Layer chicken	Legok Tanggerang	2003	+	+	+	+

Layer chicken	Legok Tanggerang	2003	+	+	+	+

Layer chicken	Bogor	2004	+	+	+	+

Native chicken	Tasikmalaya	2005	+	+	+	+

Native chicken	Tasikmalaya	2005	+	+	+	+

Native chicken	Tasikmalaya	2005	+	+	+	+

Duck	Sukabumi	2006	+	+	+	+

Stray cat	Bogor	2007	+	+	+	+

Layer Chicken	Sukabumi	2008	-	+	+	-

Native chicken	Maros South Sulawesi	2009	+	+	+	+

## Discussion

Point-of care rapid tests are designed and marketed for use in the ambulatory setting, in order to guide physicians in making the best possible clinical decisions and help farmers in preventing animal disease spreading and stopping the immediate threat to human health. Conventional methods for H5 virus detection are time-consuming and technically demanding, and most importantly, these methods are not practical for field investigation [17]. Several rapid diagnostic kits for the detection of H5 subtype viruses have been reported. But more than a couple of monoclonal antibodies or polyclonal antibodies are required to reach appropriate specificity and sensitivity of detection, which increases production cost [14]. The application of the complementary Mab pair reported in this study provides a solution to this and makes it possible for the cost effective production of rapid H5 tests for field usage.

One of the H5 strains from chicken, which can not be detected by the dot ELISA, was subjected to HA sequencing. The sequence result indicated that multiple deletions occur in its H5 sequence, such as the 353^rd ^and the 387^th ^nucleotide. These mutations may cause changes in HA protein structure and abolish the interaction to specific Mabs. These nature virus mutants may not replicate properly and spread efficiently due to their genetic instability. Therefore, it is concluded that the dot ELISA performed here is able to detect those circulating H5N1 viruses that did not change genetically in their HA genes. Unlike chicken, duck and other water fowls do not show any symptom even if they are infected with high concentration of H5 virus [18]. These virus carriers can cause virus shedding and spreading. Virus titration studies indicate that these non-symptomatic birds can shed more than 10^8 ^EID_50_/ml of the virus to the environment. The dot test developed here is sensitive enough to achieve specific early detection in poultry species. Therefore, the use of the H5 dot ELISA rapid test on site will reduce the risk of the false negative results via symptom observation only. Though, as a common challenge for all the rapid field tests, there is the possibility of false negative results due to the limitation of test sensitivity, this H5 dot ELISA serves as an effective tool for H5 screening at the very early stage. For those possible infected populations, it is still necessary to confirm with RT-PCR after the primary H5 infection screening with this rapid test first, if the clinical condition allows.

Selection of the H5 HA specific MAbs for the development of the H5 dot ELISA was based on detailed analyses of their binding properties. The selected H5-specific MAb 4C2 and 6B8 demonstrated high specificity to H5 HA in the HI assay and IFA. Neutralization escape mutants with the Mab pair showed substitutions at amino acid positions 155 and 189. These amino acids are two of the key residues in the H5 receptor-binding site of the globular head of the HA molecule. H5N1 HPAI viruses are classified into distinct phylogenic clades based on their phylogenetic divergence [19]. The MAb pair described here recognizes multiple clades of H5N1 viruses, including clades 0, 1, 2.1, 2.2, 2.3, 4, 7, and 8 in the H5 dot ELISA. This result could suggest that the epitope-binding sites of the two complementary MAbs are highly conserved in H5N1 viruses. Such conformational epitopes in the receptor binding site are HA subtype-specific [20]. Future studies will be performed to apply this Mab pair for therapeutic purpose against H5 influenza infection without mutant evasion [21]. 38 non-H5 subtype influenza virus strains were tested to be negative in this dot ELISA. Though they constitute only a small subset of the possible viruses, the cross-reactivity of the H5 dot ELISA with other subtype viruses is believed to be extremely low. Further evaluation, however, will be performed with more samples, especially human samples, to determine the specificity of the assay in a more quantitative way.

The performance of the H5 dot ELISA has been proved to be stable based on a standard method in which the kits were stored at 37°C for a week (data not shown). The study indicated that the H5 dot ELISA developed here is suitable for the usage in field where the storage condition at low temperature is not available. It has been studied as well that the performance of the test will not be affected by those potential chemical ingredients in oral or nasal swabs, such as antibiotics, mouth wash and nasal sprays. However, the only drawback of the current test is the potential cross reaction with bloody samples, which may cause false-positive results during testing. Fortunately, a new technology developed recently provides solution to this problem. The target can be detected by fluorescence labeled antibodies and be observed with a portable fluorescence reader. Any false positive signal from blood will be eliminated by using fluorescence with target-specific wave length.

## Conclusions

In conclusion, the H5 dot ELISA developed can serve as rapid devices for the on-site detection of H5 influenza virus. It has been evaluated in this study with tracheal swabs from avian species. It could also be used to test other types of swabs from other species, such as mammals. Future studies will be performed to confirm this. Based on complementary Mabs, the test can respond to more than 99% of circulating H5 influenza viruses with the as sensitivity as a rapid field test. Further investigation, however, is needed to shorten the processing time of each test for a new generation of rapid field tests.

Based on all these studies, indicating that the test is convenient for field use, this H5 Dot ELISA can be used for on-site detection of H5N1 infection in clinical or environmental specimens and facilitate the investigation of H5N1 influenza outbreaks and surveillance in poultry.

## Methods

### Viruses and cells

As shown in Table 9, twenty-four human H5N1 influenza strains (clade 2.1) isolated from Indonesia were obtained from the Ministry of Health, Indonesia. Twelve avian H5N1 influenza strains isolated from Indonesia were collected by the faculty of veterinary medicine, Bogor agriculture university, Indonesia. Forty-six H5 influenza strains were tested in Wantai biotechnology company, China. Five non-H5 subtype strains were obtained from the Agri-Food and Veterinary Authority of Singapore. Sixteen H1N1, six H3N2, and four influenza B virus strains were isolated from human clinical samples by the Department of Pathology, Singapore General Hospital. The remaining H5 and non H5 influenza viruses were generated with reverse genetics in our lab as described previously [22]. All of HA and NA genes were synthesized by GenScript. The reassortant viruses were rescued by transfecting plasmids containing HA and NA together with the remaining six gene plasmids derived from A/Puerto Rico/8/34 (H1N1) into a coculture of 293T and MDCK cells. All of H5N1 and non-H5N1 strains studied in the laboratory in Singapore are listed in Table 5 and 6. Viruses were inoculated into the allantoic cavities of 11-day-old embryonated chicken eggs and harvested following 48 h of incubation at 37°C. Virus titers were determined using hemagglutination assays according to standard methods [19]. H5N1 subtype viruses were inactivated with formaldehyde as described previously [23]. All experiments with live H5N1 and H7N7 subtype viruses were performed in a biosafety level 3 containment laboratory in compliance with CDC/NIH and WHO recommendations and also were approved by the Agri-Food and Veterinary Authority and the Ministry of Health of Singapore.

**Table 9:** Summary of the viruses tested in this study

Source	Type	Number
MOH, Indonesia	H5N1	24

Bogor, Indonesia	H5N1	12

Wantai, China	H5	46

Reverse genetics, in house	H5N1	16

AVA, Singapore	non H5N1(one H5N2, one H5N3)	7

SGH, Singapore	non H5	26

Reverse genetics, in house	non H5	9

Total	H5	100

Total	non H5	40

MDCK cells were obtained from the American Type Culture Collection (ATCC). Cells were propagated in Dulbecco's minimal essential medium (DMEM) supplemented with 10% fetal bovine serum. Virus stocks were grown in MDCK cells in DMEM supplemented with 0.5% bovine serum albumin (BSA) and 200 ng/ml of trypsin.

### Preparation and purification of Mabs

Hybridomas secreting specific Mabs were derived from BALB/c mice which had been immunized twice intramuscularly with purified H5N1 AIV in 0.1 ml of PBS, emulsified with an equal volume of adjuvant (SEPPIC, France). An intraperitoneal booster of the same dose of H5N1 virus was given three days before splenocytes were fused to the SP/2.0 myloma cells, as previously described [24]. 6B8 was generated from mice immunized with H5N2 strain A/chicken/Singapore/98, while 4C2 was obtained from mice with H5N1 strain A/human/Indonesia/CDC669/06. Hybridoma culture supernatants were screened by immunofluorescent assays using mock-infected or variant H5N1 infected MDCK as antigen, respectively, as described below. Hybridomas identified to produce specific antibody, were cloned by limiting dilution and expanded in 75 cm^2 ^flasks. One week later, the hybridoma suspension was harvested and cell debris pelleted by centrifugation at 400 g for 10 min, followed by collection of the supernatant and storage at -20°C. IgM were purified from clarified Mab supernatant using protein A affinity column (Sigma, USA) and Immnopure^® ^IgM purification kit (Pierce, IL, USA) in accordance with manufacturer's instructions. IgM concentrations were determined spectrophotometrically (Nanodrop, DE, USA).

### Hemagglutination-inhibition (HI) test

Mab 4C2 and 6B8 were subjected to HI test which was carried out according to the standard method [19]. Briefly, receptor-destroying enzyme-treated sera were serially diluted (twofold) in V-bottom, 96-well plates and mixed with an equal volume of virus. Plates were incubated for 30 min at room temperature, and 1% chicken red blood cell was added to each well. The HI endpoint was the highest serum dilution in which agglutination was not observed.

### Selection of escape mutants

Generation of escape mutants follows the standard method as described previously [21,25,26]. Serial 10-fold dilutions of A/Indonesia/CDC669/06 (H5N1) virus were mixed with an excess amount of 4C2 MAb (1 ug/ul) in an equal volume, and A/Vietnam/1203/04 (H5N1) with 6B8, and incubated at room temperature for 30 min. The mixture was inoculated into 11-day old embryonated chicken eggs. The eggs were incubated at 37°C for 48 h. Virus was harvested and used for cloning in limiting dilution in embryonated chicken eggs and the escape mutants were plaque purified. Viral RNA was isolated using LS Trizol reagent (Invitrogen) as specified by the manufacturer. Reverse transcription and PCR were performed with specific primers for the HA gene of H5 subtypes. Mutations in a HA gene were then identified by sequencing and compared with the sequence of the parent virus.

### H5 Antigen capture ELISA

96-well, round-bottom microtiter plates (Nunc, Roskilde, Demark) were coated with 1 ug/well of capture MAb in 100 ul of carbonate buffer (73 mM sodium bicarbonate and 30 mM sodium carbonate, pH 9.7) overnight at 4°C or 37°C for 2 h. The plates were washed twice with PBST, followed by two washes with PBS after each incubation with antibody or antigen. The antibody-coated plates were blocked by incubation with 100 ul of blocking buffer (PBS containing 5% milk) for 1 h at room temperature and then incubated at 37°C for 1 h with 100 ul of virus-containing samples diluted in PBST. Virus binding was detected by incubation for 1 h at 37°C with 100 ul of horseradish peroxidase-conjugated detection MAb (800 ng) (in-house labeling; Roche). Chromogen development was mediated by the addition of 100 ul of freshly prepared substrate solution (o-phenylenediamine-dihydrochloride; Sigma). The reaction was stopped by adding 0.1 N sulfuric acid, and the optical density at 490 nm was recorded. The detection limit was determined by the optical density value that gave a signal-to-noise ratio of 3.

### Dot ELISA

The dot ELISA rapid test kit with the two complementary Mabs was manufactured by Wantai biotechnology company, China [14]. The dot ELISA test was performed following the manufacturer's protocol. Briefly, 200 ul of samples was lysed with 400 ul lysis buffer and loaded on a filter device. The filtrated samples went through the membrane coated with Mabs. Following washing with wash buffer of three times, the substrate reagent was added on the membrane and the signal was developed. Results were read within 5 minutes after adding stop solution.

### Preparation of tracheal swab samples

200 samples of tracheal swab were collected from fresh avian species from Bogor and Makassar (South Sulawesi) to detect any possible existence of H5 avian influenza virus. A serial dilution (multiple of 10) was performed on the virus of subtype H5N1 with predetermined titer level. The multiplication level of the dilution started initially at 10^-1 ^and gradually increased to 10^-4^. The dissolved viruses were tested by dot ELISA kit to determine the capability of detecting the most dissolved virus in swabs. The experiment has been repeated three times. Using Reed and Muench mathematical technique, the infectivity titer of each sample was expressed as EID_50_/ml.

### RT-PCR

Extraction of total RNA was performed following manufacturers' protocol from QIAamp Viral RNA Mini Kit (Qiagen, Germany) using all necessary safety precautions. The resultant RNA was dissolved in 20 ul of RNase-free water. Three PCRs were performed using two H5 primer pairs and HA2 specific primers individually. One pair of H5 primers consist of primers J3 and B2a as described previously [27]. The primer pair is as follows: J3: GAT AAA TTC TAG CAT GCC ATT CC B2a: TTT TGT CAA TGA TTG AGT TGA CCT TAT TGG. The second H5 specific primer pair was forward primer: 5'-TCAGATTTGCATTGGTTACC-3' and reverse primer: 5'- ACTATGTAAGACCATTCCGG3'). HA2 primers were: forward primer: 5'-ACTATGAAGAATGAAACACCT-3' and reverse primer: 5' GCAATGAAATTTCCATTACTCTC-3').

One step RT-PCR cycling conditions were 60°C for 1 min, 42°C for 10 min, 50°C for 30 min, and 94°C for 15 min followed by 35 cycles of 94°C for 30 sec, 50°C for 30 sec, and 72°C for 1 min and lastly followed by 72°C for 10 min. The PCR products were resolved in 1.2% agarose gels with the sizes of around 312 bp- 456 bp. PCR products were further sequenced to confirm the identity of the products.

## Authors' contributions

FH characterized the Mabs epitopes and developed the ELISA and dot ELISA with the two Mabs. FH evaluated the sensitivity and specificity of the kit with the virus samples. RS and SM provided a part of virus samples and performed the studies with samples from avian specials. MG provided a part of virus samples and organized the colaboration. JK designed the study and analyse the results. All authors have read and approved the final manuscript.

## References

[B1] Fouchier RA Munster V Wallensten A Bestebroer TM Herfst S Smith D Rimmelzwaan GF Olsen B Osterhaus AD Characterization of a novel influenza A virus hemagglutinin subtype (H16) obtained from black-headed gulls J Virol 2005 79 5 2814 2822 10.1128/JVI.79.5.2814-2822.2005 15709000 PMC548452

[B2] Thontiravong A Payungporn S Keawcharoen J Chutinimitkul S Wattanodorn S Damrongwatanapokin S Chaisingh A Theamboonlers A Poovorawan Y Oraveerakul K The single-step multiplex reverse transcription-polymerase chain reaction assay for detecting H5 and H7 avian influenza A viruses Tohoku J Exp Med 2007 211 1 75 79 10.1620/tjem.211.75 17202774

[B3] Apisarnthanarak A Warren DK Fraser VJ Issues relevant to the adoption and modification of hospital infection-control recommendations for avian influenza (H5N1 infection) in developing countries Clin Infect Dis 2007 45 10 1338 1342 10.1086/522538 17968831 PMC7107968

[B4] Babakir-Mina M Balestra E Perno CF Aquaro S Influenza virus A (H5N1): a pandemic risk? New Microbiol 2007 30 2 65 78 17619249

[B5] Park AW Glass K Dynamic patterns of avian and human influenza in east and southeast Asia Lancet Infect Dis 2007 7 8 543 548 10.1016/S1473-3099(07)70186-X 17646027

[B6] Peiris JS de Jong MD Guan Y Avian influenza virus (H5N1): a threat to human health Clin Microbiol Rev 2007 20 2 243 267 10.1128/CMR.00037-06 17428885 PMC1865597

[B7] Alexander DJ Brown IH History of highly pathogenic avian influenza Rev Sci Tech 2009 28 1 19 38 19618616 10.20506/rst.28.1.1856

[B8] Organization. WH Cumulative number of confirmed human cases of avian influenza A/(H5N1) reported to WHO 2009 http://www.who.int/csr/disease/avian_influenza/country/cases_table_2010_08_31/en/index.html

[B9] Kaiser L Briones MS Hayden FG Performance of virus isolation and Directigen Flu A to detect influenza A virus in experimental human infection J Clin Virol 1999 14 3 191 197 10.1016/S1386-6532(99)00058-X 10614856

[B10] Woo PC Chiu SS Seto WH Peiris M Cost-effectiveness of rapid diagnosis of viral respiratory tract infections in pediatric patients J Clin Microbiol 1997 35 6 1579 1581 9163486 10.1128/jcm.35.6.1579-1581.1997 PMC229791

[B11] Chen J Jin M Yu Z Dan H Zhang A Song Y Chen H A latex agglutination test for the rapid detection of avian influenza virus subtype H5N1 and its clinical application J Vet Diagn Invest 2007 19 2 155 160 17402609 10.1177/104063870701900203

[B12] Wei HL Bai GR Mweene AS Zhou YC Cong YL Pu J Wang S Kida H Liu JH Rapid detection of avian influenza virus a and subtype H5N1 by single step multiplex reverse transcription-polymerase chain reaction Virus Genes 2006 32 3 261 267 10.1007/s11262-005-6910-4 16732478

[B13] Payungporn S Phakdeewirot P Chutinimitkul S Theamboonlers A Keawcharoen J Oraveerakul K Amonsin A Poovorawan Y Single-step multiplex reverse transcription-polymerase chain reaction (RT-PCR) for influenza A virus subtype H5N1 detection Viral Immunol 2004 17 4 588 593 10.1089/vim.2004.17.588 15671756

[B14] Chen Y Xu F Fan X Luo H Ge S Zheng Q Xia N Chen H Guan Y Zhang J Evaluation of a rapid test for detection of H5N1 avian influenza virus J Virol Methods 2008 154 1-2 213 215 10.1016/j.jviromet.2008.08.013 18793671

[B15] Yokoyama WM Production of monoclonal antibody supernatant and ascites fluid Curr Protoc Mol Biol 2008 Chapter 11 Unit 11.10 18633992 10.1002/0471142727.mb1110s83

[B16] Wu WL Chen Y Wang P Song W Lau SY Rayner JM Smith GJ Webster RG Peiris JS Lin T Antigenic profile of avian H5N1 viruses in Asia from 2002 to 2007 J Virol 2008 82 4 1798 1807 10.1128/JVI.02256-07 18077726 PMC2258718

[B17] Storch GA Rapid diagnostic tests for influenza Curr Opin Pediatr 2003 15 1 77 84 10.1097/00008480-200302000-00013 12544276

[B18] Zhang G Shoham D Gilichinsky D Davydov S Castello JD Rogers SO Evidence of influenza a virus RNA in siberian lake ice J Virol 2006 80 24 12229 12235 10.1128/JVI.00986-06 17035314 PMC1676296

[B19] Abdel-Ghafar AN Chotpitayasunondh T Gao Z Hayden FG Nguyen DH de Jong MD Naghdaliyev A Peiris JS Shindo N Soeroso S Update on avian influenza A (H5N1) virus infection in humans N Engl J Med 2008 358 3 261 273 10.1056/NEJMra0707279 18199865

[B20] Stevens J Blixt O Tumpey TM Taubenberger JK Paulson JC Wilson IA Structure and receptor specificity of the hemagglutinin from an H5N1 influenza virus Science 2006 312 5772 404 410 10.1126/science.1124513 16543414

[B21] Prabakaran M Prabhu N He F Hongliang Q Ho HT Qiang J Meng T Goutama M Kwang J Combination therapy using chimeric monoclonal antibodies protects mice from lethal H5N1 infection and prevents formation of escape mutants PLoS One 2009 4 5 e5672 10.1371/journal.pone.0005672 19478856 PMC2682562

[B22] Ho HT Qian HL He F Meng T Szyporta M Prabhu N Prabakaran M Chan KP Kwang J Rapid detection of H5N1 subtype influenza viruses by antigen capture enzyme-linked immunosorbent assay using H5- and N1-specific monoclonal antibodies Clin Vaccine Immunol 2009 16 5 726 732 10.1128/CVI.00465-08 19321691 PMC2681585

[B23] He Q Velumani S Du Q Lim CW Ng FK Donis R Kwang J Detection of H5 avian influenza viruses by antigen-capture enzyme-linked immunosorbent assay using H5-specific monoclonal antibody Clin Vaccine Immunol 2007 14 5 617 623 10.1128/CVI.00444-06 17344345 PMC1865641

[B24] Yokoyama WM Production of monoclonal antibody supernatant and ascites fluid Curr Protoc Mol Biol 2001 Chapter 11 Unit 11.10 10.1002/0471142727.mb1110s83 18633992

[B25] Kaverin NV Rudneva IA Ilyushina NA Varich NL Lipatov AS Smirnov YA Govorkova EA Gitelman AK Lvov DK Webster RG Structure of antigenic sites on the haemagglutinin molecule of H5 avian influenza virus and phenotypic variation of escape mutants J Gen Virol 2002 83 Pt 10 2497 2505 12237433 10.1099/0022-1317-83-10-2497

[B26] Kaverin NV Rudneva IA Govorkova EA Timofeeva TA Shilov AA Kochergin-Nikitsky KS Krylov PS Webster RG Epitope mapping of the hemagglutinin molecule of a highly pathogenic H5N1 influenza virus by using monoclonal antibodies J Virol 2007 81 23 12911 12917 10.1128/JVI.01522-07 17881439 PMC2169086

[B27] Slomka MJ Coward VJ Banks J Londt BZ Brown IH Voermans J Koch G Handberg KJ Jorgensen PH Cherbonnel-Pansart M Identification of sensitive and specific avian influenza polymerase chain reaction methods through blind ring trials organized in the European Union Avian Dis 2007 51 1 Suppl 227 234 10.1637/7674-063006R1.1 17494558

